# Deciphering the causal relationship between plasma and cerebrospinal fluid metabolites and glioblastoma multiforme: a Mendelian Randomization study

**DOI:** 10.18632/aging.205818

**Published:** 2024-05-10

**Authors:** Zhiwei Zhou, Haibin Leng

**Affiliations:** 1 Department of Neurosurgery, Changde Hospital, Xiangya School of Medicine, Central South University (The First People’s Hospital of Changde City), Changde, Hunan 415003, People’s Republic of China

**Keywords:** glioblastoma multiforme, metabolites, Trimethylamine N-oxide, Mendelian Randomization, pathogenesis

## Abstract

Background: Glioblastoma Multiforme (GBM) is one of the most aggressive and fatal brain cancers. The study of metabolites could be crucial for understanding GBM’s biology and reveal new treatment strategies.

Methods: The GWAS data for GBM were sourced from the FinnGen database. A total of 1400 plasma metabolites were collected from the GWAS Catalog dataset. The cerebrospinal fluid (CSF) metabolites data were collected from subsets of participants in the WADRC and WRAP studies. We utilized the inverse variance weighting (IVW) method as the primary tool to explore the causal relationship between metabolites in plasma and CSF and glioblastoma, ensuring the exclusion of instances with horizontal pleiotropy. Additionally, four supplementary analytical methods were applied to reinforce our findings. Aberrant results were identified and omitted based on the outcomes of the leave-one-out sensitivity analysis. Conclusively, a reverse Mendelian Randomization analysis was also conducted to further substantiate our results.

Results: The study identified 69 plasma metabolites associated with GBM. Of these, 40 metabolites demonstrated a significant positive causal relationship with GBM, while 29 exhibited a significant negative causal association. Notably, Trimethylamine N-oxide (TMAO) levels in plasma, not CSF, were found to be a significant exposure factor for GBM (OR = 3.1627, 95% CI = (1.6347, 6.1189), *P* = 0.0006). The study did not find a reverse causal relationship between GBM and plasma TMAO levels.

Conclusions: This research has identified 69 plasma metabolites potentially associated with the incidence of GBM, among which TMAO stands out as a promising candidate for an early detectable biomarker for GBM.

## INTRODUCTION

Glioblastoma Multiforme (GBM) is among the most aggressive and fatal brain cancers, known for its rapid proliferation and diffuse infiltration into surrounding brain tissue [1]. Despite advancements in neuro-oncology, the prognosis for GBM remains dire, with median survival rates typically less than 15 months post-diagnosis [2–4]. The complexity of GBM pathogenesis, alongside its resilience against conventional therapeutic regimens, underscores the critical need for novel diagnostic and therapeutic approaches [5].

Metabolomics, the extensive study of small molecules within biological systems, stands at the forefront of this challenge, offering promising potential to decipher the altered metabolic pathways associated with cancer phenotypes [6, 7]. Plasma metabolites, as downstream products of genetic, enzymatic, and cellular processes, reflect the dynamic state of GBM’s pathophysiology and therefore hold promise for revealing new facets of its biology [8–10]. The identification and in-depth analysis of these metabolites could unveil novel biomarkers that not only deepen our understanding of GBM’s underpinnings but also pave the way for targeted treatment strategies. In parallel, cerebrospinal fluid (CSF) presents a distinctive lens into the brain’s internal environment, potentially yielding critical insights into metabolic changes uniquely pertinent to neurological pathology [11–13]. Given its intimate association with the brain’s extracellular space, CSF metabolomic analysis could offer a more direct evaluation of the biochemical environment that influences GBM’s progression [14, 15].

To fully leverage the insights offered by metabolomics, the integration of genomic data from Genome-Wide Association Studies (GWAS) is essential [16]. GWAS provides a broad spectrum for identifying genetic variants that influence disease susceptibility and progression, including GBM [17–19]. By integrating GWAS findings with observed metabolic changes in both plasma and CSF, we can construct a more detailed portrait of GBM’s pathogenesis. Utilizing this integrated approach, Mendelian Randomization (MR) analysis serves as a robust tool for deducing causality from observational data [20, 21]. MR utilizes genetic variants as instrumental variables to map the cause-effect trajectories between plasma metabolites and GBM, effectively bypassing the confounding variables that often confound observational studies [22, 23].

Therefore, this study is dedicated to investigating 1,400 plasma metabolites intricately connected to GBM’s emergence and development. By pinpointing metabolites that hold a significant causal link to GBM, we seek to understand their biological significance and their viability as biomarkers or therapeutic agents. Moreover, we aim to corroborate these findings within CSF metabolites, enriching our comprehension of the metabolic framework of GBM. Through MR analysis, our research strives to shed light on the metabolic processes that could be instrumental in GBM’s oncogenesis and progression, ultimately contributing to the development of potential clinical interventions for this formidable ailment.

## MATERIALS AND METHODS

### Study design

Our approach was firmly rooted in adhering to the fundamental tenets of MR analysis. Firstly, we ensured a robust association exists between genetic variants and the exposure. Secondly, we ascertained that these genetic variants are not associated with potential confounding factors. Finally, we confirmed that the impact of genetic variants on the outcome is mediated exclusively through the exposure, without the influence of alternative biological pathways. Additionally, to reinforce the validity of our findings, a reverse MR analysis was conducted, leveraging significant results from the initial MR analysis to enhance the robustness of the outcomes.

**Figure 1:**
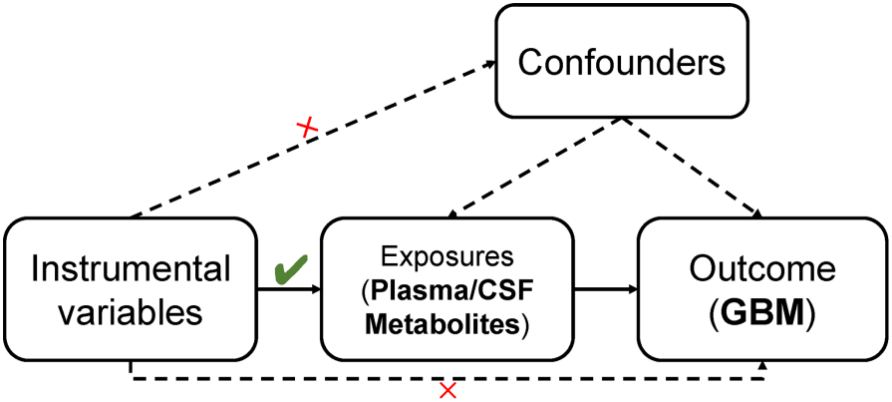
The whole MR study design.

The study’s specific design and methodology are comprehensively illustrated in Figure 1. In our Mendelian Randomization analysis study, we employed the application of the Inverse Variance Weighted (IVW) method as our primary tool for analyzing a wide array of 1,400 plasma metabolites. The objective was to identify those metabolites that act as potential exposure factors influencing the pathogenesis of GBM. Following this initial identification, we delved deeper to ascertain the most significant causal relationships between these plasma metabolites and GBM, focusing on determining the extent of their impact on GBM development. A critical component of our study involved examining the possibility of reverse causation – whether the presence of GBM could influence the levels of these key plasma metabolites, thereby exploring the bidirectional nature of their interactions. The investigation was further extended to include CSF, aiming to identify and compare the causal relationships of similar metabolites within CSF with GBM.

### GWAS data sources of GBM

For our study, the GWAS dataset specific to Glioblastoma Multiforme was sourced from the FinnGen database (https://www.finngen.fi). The dataset originated from a detailed GWAS conducted on a cohort of European descent, comprising 243 individuals diagnosed with GBM, contrasted against a control group of 287,137 participants. In this comprehensive analysis, approximately 16.38 million genetic variants were meticulously examined. Each variant underwent rigorous quality control measures and was processed using advanced imputation methods.

### Plasma metabolites GWAS data collection

The collection of GWAS data for 1,400 plasma metabolites was conducted through a comprehensive approach, utilizing several datasets [24]. We sourced the GWAS summary statistics from the GWAS Catalog, available at EBI GWAS Catalog. This included a wide array of studies, categorized under specific accession numbers: GCST90199621 to GCST90201020 for European GWASs and GCST90201021 to GCST90204063 for non-European GWASs. In addition to these aggregated statistics, individual-level data for certain metabolites were obtained from the Canadian Longitudinal Study on Aging (CLSA), accessible at CLSA, where the data availability was contingent upon meeting stringent criteria for accessing de-identified CLSA data. This was in adherence to ethical standards and data privacy regulations. To further ensure the accuracy and pertinence of our metabolite analysis, we cross-referenced our findings with the Human Metabolome Database (HMDB). The assembly of these extensive and diverse range of GWAS data forms the bedrock of our rigorous Mendelian Randomization analysis, focusing on exploring the relationships between plasma metabolites and GBM.

### CSF metabolites GWAS data collection

Our research utilized GWAS summary datasets (https://gwas.mrcieu.ac.uk) focusing on cerebrospinal fluid metabolites. These datasets were derived from a subset of participants in the Wisconsin Alzheimer’s Disease Research Center (WADRC) and the Wisconsin Registry for Alzheimer’s Prevention (WRAP) studies. In these studies, CSF samples were collected via lumbar punctures (LPs). The collection and storage protocols for these samples were uniform across both studies, as described in prior publication [25]. The procedure involved collecting fasting CSF samples from participants in the morning, which were then mixed, centrifuged, aliquoted, and stored at −80°C. Metabolon performed an untargeted metabolomics analysis of these samples using Ultrahigh Performance Liquid Chromatography-Tandem Mass Spectrometry (UPLC-MS/MS), processing samples from both WADRC and WRAP on the same platform for consistency. In this analysis, 412 metabolites were quantified, with 354 identified and 58 of unknown structural identity. The study included 689 participants in total, with 532 from WADRC and 168 from WRAP, each providing unique CSF samples for metabolite analysis. The WADRC participants were selected based on criteria such as being aged ≥ 45, having decisional capacity, and the ability to fast for 12 hours. Exclusion criteria encompassed a history of specific medical conditions like kidney dysfunction, congestive heart failure, major neurologic disorders (excluding dementia), and others. This study was a part of the Generations of WRAP (GROW) study and received approval from the University of Wisconsin Health Sciences Institutional Review Board.

### Instrumental variable selection

In line with established methodologies in contemporary research, we selected instrumental variables (IVs) for each plasma, CSF metabolites, and GBM using a significance threshold of 1 × 10^−5^. The SNP clumping process, instrumental in reducing linkage disequilibrium (LD), was carried out utilizing PLINK software. This step involved setting an LD r^2^ threshold of less than 0.1 within a 500 kilobase (kb) window, referencing data from the 1000 Genomes Project, which is a methodological choice that reflects a careful consideration of the trade-offs between specificity, sensitivity, and computational feasibility in the context of genetic association studies. To evaluate the effectiveness of each IV, we calculated both the proportion of phenotypic variation explained (PVE) and the F statistic, with a focus on excluding weak instruments that could potentially skew the analysis. This study retained suitable IVs for further analysis, following the removal of those with F statistics falling below 10.

### Mendelian Randomization analysis

In our study, statistical analyses were performed using the R programming environment, specifically employing the “TwoSampleMR” package (version 0.5.7). This package facilitated the application of several MR methodologies, encompassing IVW, weighted median, mode-based estimation, MR-Egger, Simple mode, and Weighted mode approaches. These methods were pivotal in establishing the causal relationships between identified exposures and outcomes. To evaluate the heterogeneity among instrumental variables (IVs), we utilized Cochran’s Q statistic alongside its corresponding *p*-values. Where significant heterogeneity was identified, we opted for a random-effects model within the IVW method to accommodate variability across different IVs. The MR-Egger regression method was integral in assessing the presence of horizontal pleiotropy, with a focus on the intercept; a non-zero value was indicative of such pleiotropy. Additionally, we employed the MR-PRESSO technique for the identification and exclusion of outliers potentially exhibiting horizontal pleiotropy. Our analytical process also included the generation of scatter and funnel plots. These visual representations were crucial in verifying the consistency and robustness of our causal findings, thereby enhancing the reliability of our conclusions drawn from the MR analyses.

## RESULTS

### Mendelian Randomization analysis of 1,400 plasma metabolites’ impact on GBM

In this study, we conducted a comprehensive MR analysis, focusing on 1,400 plasma metabolites as exposure factors. The analysis primarily utilized the IVW method, rigorously adjusting for linkage disequilibrium and potential confounders. Our objective was to elucidate metabolites critically involved in the pathogenesis and progression of GBM. As delineated in Table 1, among 69 plasma metabolites showing potential causal associations with GBM (*p* < 0.05), Trimethylamine N-oxide levels were identified as the most significant factor potentially exacerbating GBM progression (OR = 3.1627, 95% CI = (1.6347, 6.1189), *P* = 0.0006). Following in significance was Erucate (22:1n9), exhibiting an OR of 2.7360 and a 95% CI from 1.4258 to 5.2502 (*P* = 0.0025). On the contrary, Cysteine S-sulfate levels appeared to demonstrate a notable protective effect, with an OR of 0.4536 and a 95% CI of (0.2461, 0.8359) (*P* = 0.0113).

**Table 1:** Results of two-sample Mendelian Randomization analysis assessing the impact of 1,400 plasma metabolites on GBM, primarily utilizing inverse variance weighted methods.

**ID**	**ReportedTrait**	**Sample**	**Ancestry**	**OR**	**95L OR**	**95H OR**	***p*-value**
**GCST90199920**	**Trimethylamine n-oxide levels**	**8218**	**European**	**3.1627**	**1.6347**	**6.1189**	**0.0006**
GCST90200284	Erucate (22:1n9) levels	8208	European	2.7360	1.4258	5.2502	0.0025
GCST90200662	X-25790 levels	8221	European	2.4867	1.2349	5.0074	0.0107
GCST90200528	X-17146 levels	7778	European	2.2010	1.2886	3.7595	0.0039
GCST90199677	3-methyl-2-oxobutyrate levels	8254	European	2.1938	1.2298	3.9136	0.0078
GCST90200173	Glutamine conjugate of C6H10O2 (2) levels	8233	European	2.1883	1.1261	4.2523	0.0209
GCST90199765	4-hydroxyhippurate levels	8259	European	2.1799	1.2588	3.7750	0.0054
GCST90199658	Alpha-hydroxyisocaproate levels	8250	European	2.1198	1.2361	3.6351	0.0063
GCST90200843	Adenosine 5′-monophosphate (AMP) to flavin adenine dinucleotide (FAD) ratio	6188	European	2.0472	1.2128	3.4558	0.0073
GCST90200060	1-(1-enyl-palmitoyl)-2-linoleoyl-GPC (p-16:0/18:2) levels	8260	European	1.9522	1.1441	3.3311	0.0141
GCST90199985	Methyl-4-hydroxybenzoate sulfate levels	8285	European	1.8924	1.1424	3.1347	0.0133
GCST90199831	1-stearoyl-2-oleoyl-GPE (18:0/18:1) levels	8273	European	1.8317	1.2630	2.6567	0.0014
GCST90199988	Tyramine O-sulfate levels	7254	European	1.8267	1.1027	3.0262	0.0193
GCST90200037	1-stearoyl-2-linoleoyl-gpc (18:0/18:2) levels	8231	European	1.7909	1.1086	2.8933	0.0173
GCST90199865	2-hydroxyglutarate levels	7769	European	1.7890	1.1744	2.7254	0.0068
GCST90200328	3-methoxytyrosine levels	8258	European	1.7788	1.1579	2.7326	0.0086
GCST90200968	Phosphate to 5-oxoproline ratio	8217	European	1.7612	1.2374	2.5069	0.0017
GCST90200140	Carotene diol (2) levels	8196	European	1.7488	1.1078	2.7607	0.0164
GCST90200807	Carnitine to ergothioneine ratio	8110	European	1.7001	1.0482	2.7575	0.0315
GCST90200454	Kynurenate levels	8241	European	1.6522	1.1387	2.3973	0.0082
GCST90199664	Phenyllactate (PLA) levels in elite athletes	8235	European	1.6438	1.0680	2.5301	0.0239
GCST90200080	1-palmitoyl-2-oleoyl-GPI (16:0/18:1) levels	8123	European	1.6274	1.0701	2.4749	0.0228
GCST90200281	Phosphoethanolamine levels	8253	European	1.6239	1.0747	2.4537	0.0213
GCST90200371	3-(4-hydroxyphenyl)lactate levels	8259	European	1.6192	1.0070	2.6035	0.0467
GCST90200883	Phosphate to acetoacetate ratio	6791	European	1.6149	1.0585	2.4639	0.0262
GCST90200198	2-hydroxysebacate levels	7865	European	1.6146	1.0041	2.5964	0.0481
GCST90199794	1-oleoyl-GPE (18:1) levels	8283	European	1.6144	1.0319	2.5256	0.0359
GCST90200967	Phosphate to urate ratio	8204	European	1.5890	1.0299	2.4517	0.0364
GCST90200346	4-acetaminophen sulfate levels	4197	European	1.5413	1.0477	2.2674	0.0280
GCST90199732	1-stearoyl-gpc (18:0) levels	8240	European	1.5352	1.0478	2.2493	0.0278
GCST90199673	DHEAS levels	8228	European	1.5243	1.0470	2.2192	0.0278
GCST90200331	1-palmitoyl-2-oleoyl-GPE (16:0/18:1) levels	8262	European	1.5240	1.1272	2.0603	0.0062
GCST90200839	Adenosine 5′-diphosphate (ADP) to aspartate ratio	4563	European	1.4796	1.0996	1.9909	0.0097
GCST90199772	1-stearoyl-GPE (18:0) levels	8270	European	1.4644	1.0411	2.0597	0.0284
GCST90200144	Dihomo-linoleoylcarnitine (C20:2) levels	7887	European	1.4366	1.0226	2.0181	0.0367
GCST90200082	1-oleoyl-2-linoleoyl-GPE (18:1/18:2) levels	8205	European	1.4132	1.0659	1.8736	0.0162
GCST90200603	X-23641 levels	6673	European	1.3989	1.0215	1.9157	0.0364
GCST90200039	1-stearoyl-2-linoleoyl-GPE (18:0/18:2) levels	8241	European	1.3935	1.0262	1.8923	0.0335
GCST90199844	1-palmitoyl-2-linoleoyl-GPE (16:0/18:2) levels	8266	European	1.3276	1.0248	1.7199	0.0319
GCST90200054	1-palmitoyl-2-arachidonoyl-GPE (16:0/20:4) levels	8257	European	1.3265	1.0027	1.7549	0.0478
GCST90199660	1,2-dipalmitoyl-gpc (16:0/16:0) levels	8264	European	0.7126	0.5125	0.9910	0.0440
GCST90200014	3beta-hydroxy-5-cholestenoate levels	8259	European	0.6775	0.4642	0.9887	0.0435
GCST90200608	X-22162 levels	8249	European	0.6746	0.4729	0.9623	0.0299
GCST90200799	Spermidine to adenosine 5′-diphosphate (ADP) ratio	4195	European	0.6723	0.4520	0.9999	0.0500
GCST90200078	1-myristoyl-2-arachidonoyl-GPC (14:0/20:4) levels	8264	European	0.6607	0.4567	0.9560	0.0279
GCST90200788	5-oxoproline to citrate ratio	8256	European	0.6537	0.4380	0.9757	0.0375
GCST90200500	X-12816 levels	5190	European	0.6478	0.4604	0.9115	0.0127
GCST90199912	N-oleoyltaurine levels	7375	European	0.6441	0.4257	0.9744	0.0373
GCST90199888	Cinnamoylglycine levels	7824	European	0.6357	0.4048	0.9985	0.0492
GCST90200116	Ceramide (d18:1/14:0, d16:1/16:0) levels	7270	European	0.6217	0.4184	0.9240	0.0187
GCST90200935	Carnitine to propionylcarnitine (C3) ratio	8185	European	0.6215	0.4062	0.9510	0.0284
GCST90200802	Histidine to pyruvate ratio	8241	European	0.6157	0.4144	0.9147	0.0163
GCST90200090	Dihomo-linolenoyl-choline levels	6954	European	0.6141	0.3777	0.9985	0.0493
GCST90200873	Cortisol to 4-cholesten-3-one ratio	7027	European	0.6097	0.3801	0.9780	0.0402
GCST90200349	Eicosapentaenoate (EPA; 20:5n3) levels	8257	European	0.6053	0.4108	0.8919	0.0111
GCST90200620	X-24243 levels	7540	European	0.5927	0.3570	0.9841	0.0432
GCST90200874	Cysteinylglycine to taurine ratio	8193	European	0.5869	0.3684	0.9349	0.0249
GCST90199983	5alpha-androstan-3alpha,17beta-diol monosulfate (2) levels	5920	European	0.5756	0.3590	0.9227	0.0218
GCST90200846	Phenylalanine to phosphate ratio	8294	European	0.5740	0.3537	0.9315	0.0246
GCST90200978	Caffeine to paraxanthine ratio	7873	European	0.5389	0.3489	0.8324	0.0053
GCST90200483	X-12216 levels	8181	European	0.5330	0.3296	0.8618	0.0103
GCST90200100	Perfluorooctanesulfonate (PFOS) levels	8218	European	0.5296	0.3460	0.8107	0.0034
GCST90200944	Alpha-ketoglutarate to pyruvate ratio	8272	European	0.5290	0.2813	0.9950	0.0482
GCST90199801	Hydroquinone sulfate levels	7682	European	0.5164	0.2932	0.9095	0.0221
GCST90200367	3-Hydroxybutyrate levels	8292	European	0.5109	0.2641	0.9884	0.0461
GCST90200043	1-palmitoyl-2-docosahexaenoyl-gpc (16:0/22:6) levels	8256	European	0.4961	0.3083	0.7982	0.0039
GCST90200727	Adenosine 3′,5′-cyclic monophosphate (cAMP) to taurocholate ratio	6329	European	0.4906	0.2794	0.8617	0.0132
GCST90200221	Glycoursodeoxycholic acid sulfate (1) levels	6622	European	0.4882	0.3176	0.7506	0.0011
GCST90199671	Cysteine s-sulfate levels	8229	European	0.4536	0.2461	0.8359	0.0113

### Further investigation into the causal association between Trimethylamine N-oxide levels and GBM

**Figure 2:**
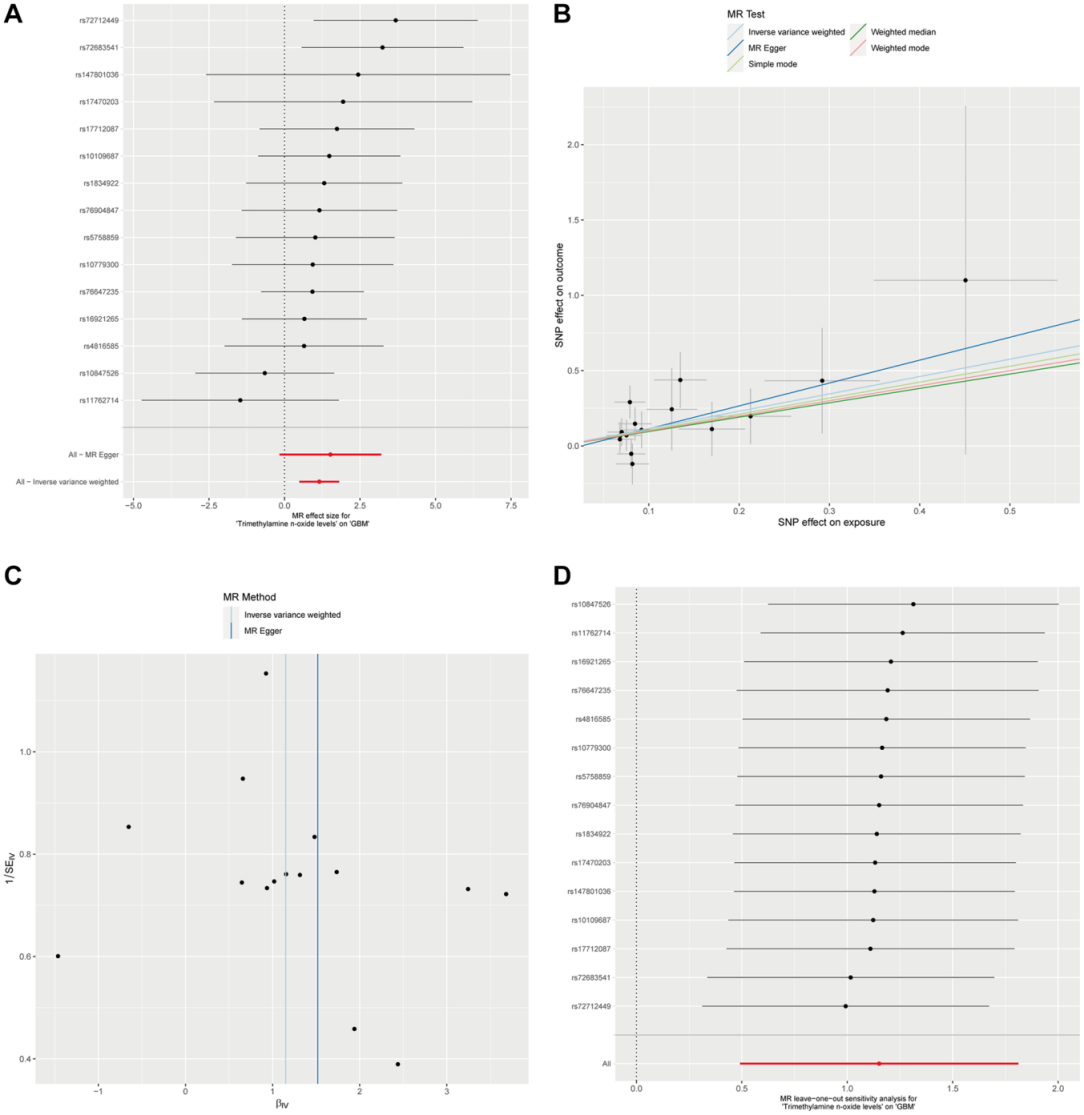
**The causal association between plasma Trimethylamine N-oxide and GBM.** (**A**). Forest plot illustrating ORs and 95% CIs for the association, with individual SNPs represented by black squares and their CIs by horizontal lines; the pooled OR is indicated by a red line. (**B**) Scatter plot showing SNP effects on TMAO levels versus GBM risk, with lines for different MR methods showing the causal relationship’s direction and strength. (**C**) MR-Egger intercept test assessing horizontal pleiotropy, where the x-axis intercept value indicates pleiotropy presence, and the I² statistic on the y-axis shows instrumental variable heterogeneity; a vertical blue line shows the null intercept, suggesting no pleiotropy. (**D**) Leave-one-out sensitivity analysis indicating the stability of the causal estimate across SNPs, with a consistent overall estimate shown by a red line, suggesting no individual SNP significantly alters the MR estimate.

Considering the significant positive causal relationship identified between plasma Trimethylamine N-oxide levels and the development of GBM, we conducted a detailed examination of this association. To encompass a broader set of instrumental variables (IVs), we employed a relaxed significance threshold of *p* < 1 × 10^−5^. This adjustment led to the inclusion of 15 single nucleotide polymorphisms (SNPs) in our analysis. Utilizing the IVW method, complemented by the Weighted median approach, our findings affirm a significant causal relationship between Trimethylamine N-oxide levels and GBM, as evidenced by the P-values below the threshold of 0.05 (Figure 2A, 2B, and Supplementary Table 1). Moreover, our analyses did not detect any evidence of heterogeneity or horizontal pleiotropy, further substantiating the robustness of our results (Supplementary Table 1). To rigorously evaluate the robustness of our MR findings, we performed heterogeneity tests and conducted a leave-one-out sensitivity analysis. The MR-Egger intercept test, as depicted in Figure 2C, was utilized to assess the presence of horizontal pleiotropy among the instrumental variables. The results indicated by the intercept close to zero and the low I² statistic suggest that there is no significant horizontal pleiotropy affecting our MR analysis. Figure 2D illustrates the results from the leave-one-out sensitivity analysis. We observed that the omission of no single SNP resulted in a substantial change in the overall causal estimate. The consistency of the effect estimates across this analysis further confirms the stability and reliability of our main findings, indicating that our MR analysis is not driven by any single SNP and that the association between plasma Trimethylamine N-oxide levels and GBM is not an artifact of any individual genetic variant used as an instrument. These analyses strengthen the evidence for a causal relationship between plasma Trimethylamine N-oxide levels and GBM progression.

### Investigating the causal effect of GBM on plasma Trimethylamine N-oxide levels

**Figure 3:**
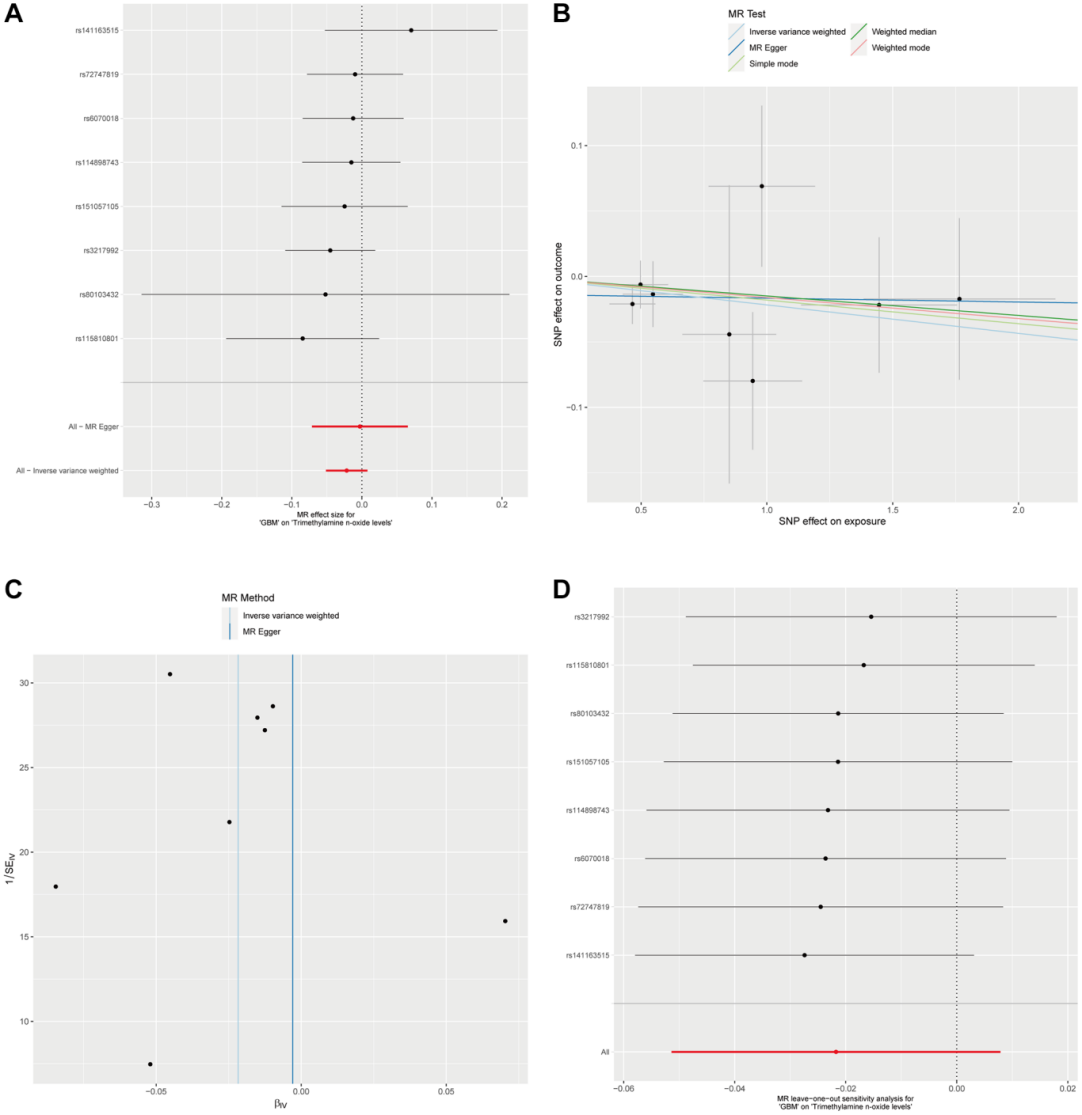
**Analysis of GBM Impact on plasma Trimethylamine N-oxide levels via reverse MR.** (**A**). Forest plot for the reverse MR analysis of GBM’s effect on Trimethylamine N-oxide levels. (**B**) This scatter plot maps the SNP effects on GBM against their effects on plasma Trimethylamine N-oxide levels, applying various MR methods. (**C**) Displayed is the MR-Egger intercept test for the reverse MR analysis. The proximity of the intercept to the zero line and the lack of deviation indicates no evidence of horizontal pleiotropy in the analysis. (**D**) The leave-one-out sensitivity analysis for the reverse MR is shown here, demonstrating the effect size stability when each SNP is sequentially excluded.

In a subsequent investigation to determine the potential influence of GBM on plasma Trimethylamine N-oxide levels, we conducted a reverse MR analysis. By setting a significance threshold of *p* < 1 × 10^−5^, we obtained 8 SNPs as instrumental variables for our reverse MR framework. Utilizing the Inverse Variance Weighted method in conjunction with four additional MR methodologies, it did not show a significant causal impact of GBM on plasma Trimethylamine N-oxide levels, with all P-values exceeding the 0.05 threshold (Figure 3A and Supplementary Table 2). Additionally, in the reverse analysis where GBM was posited as the exposure and plasma Trimethylamine N-oxide levels as the outcome, we found no substantial evidence of heterogeneity or horizontal pleiotropy (Supplementary Table 2). The collective insights from our MR analyses firmly suggest the absence of a causal relationship between GBM as an exposure factor and plasma Trimethylamine N-oxide levels as an outcome. This comprehensive approach underlines the one-directional nature of the association, where plasma Trimethylamine N-oxide levels potentially influence GBM risk but not vice versa (Figure 3A–3D).

### Exploration of cerebrospinal fluid Trimethylamine N-oxide levels in GBM causality

**Figure 4:**
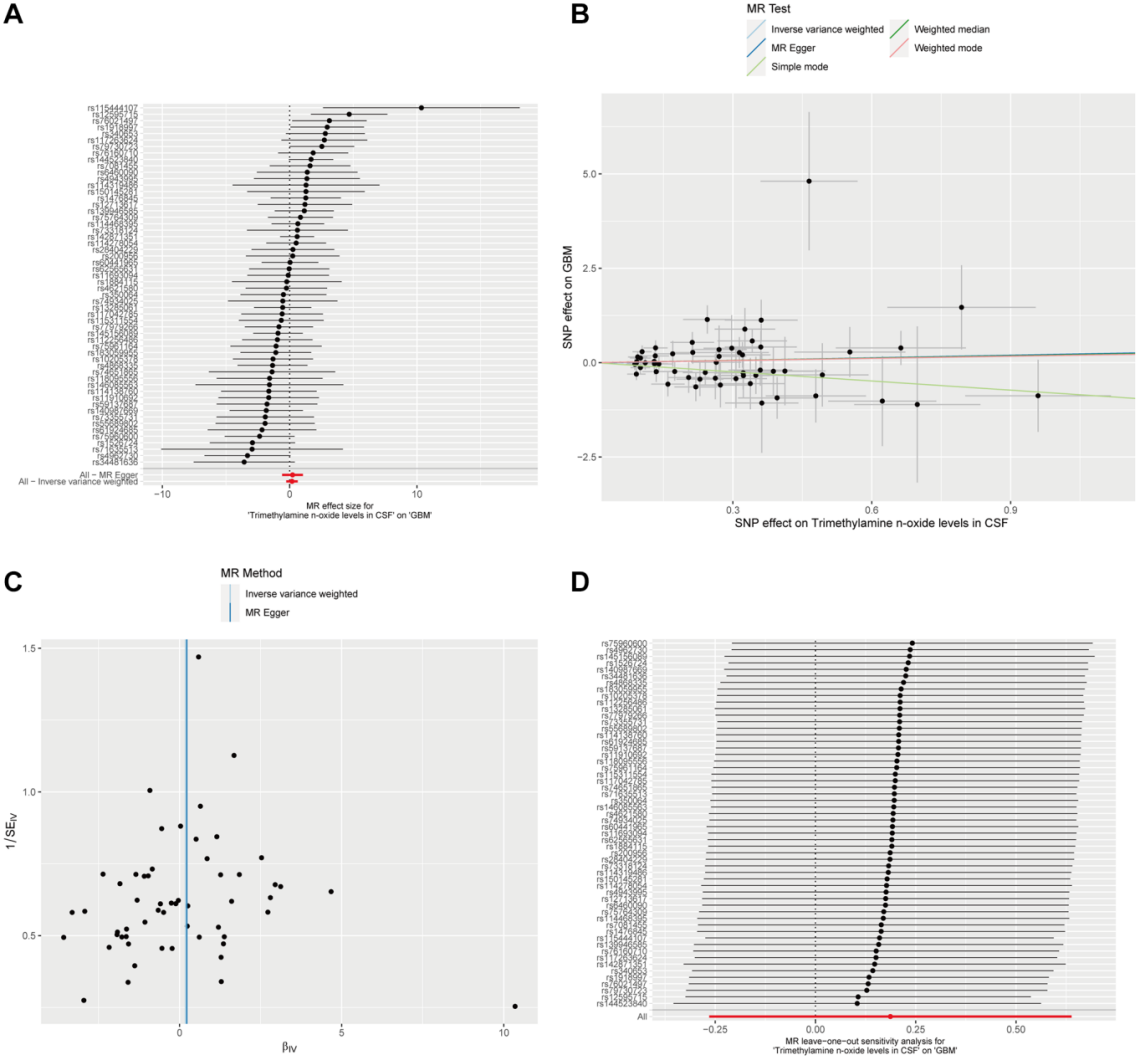
**Mendelian Randomization analysis of CSF Trimethylamine N-oxide levels and GBM.** (**A**) The forest plot displays the estimated effects of individual SNPs of CSF Trimethylamine N-oxide levels on the risk of GBM. (**B**) The scatter plot maps the effects of SNPs on Trimethylamine N-oxide levels in CSF against their influence on GBM. (**C**) The MR-Egger intercept test for pleiotropy indicates the intercepts near zero, implying no significant horizontal pleiotropy that would undermine the validity of the instrumental variables used. (**D**) A leave-one-out sensitivity analysis shows stability in the causal estimation; removing individual SNPs does not alter the overall inference, as indicated by the clustering around the null line, underscoring the non-causal relationship between CSF Trimethylamine N-oxide levels and GBM.

In an extension of our investigation into potential causal factors for GBM, we explored whether Trimethylamine N-oxide levels in CSF could also serve as an influencing factor for the development and progression of GBM. Utilizing the ebi-a-GCST90026279 dataset, we analyzed 291 European CSF samples. A significance threshold of *p* < 1 × 10^−5^ allowed us to identify 56 SNPs as instrumental variables for CSF Trimethylamine N-oxide Levels. Our analysis, employing the Inverse Variance Weighted method alongside four additional MR methodologies, did not demonstrate a significant causal effect of Trimethylamine N-oxide levels in CSF on GBM. All P-values were above the 0.05 threshold, indicating no statistically significant causal relationship (Figure 4A, 4B and Supplementary Table 3). Furthermore, we observed no substantial evidence of heterogeneity or horizontal pleiotropy in these analyses (Supplementary Table 3 and Figure 4C, 4D). These collective findings from our MR analyses imply that there is no causal link between Trimethylamine N-oxide levels in CSF as an exposure factor and GBM as an outcome.

## DISCUSSION

The findings of this study represent a potential advancement in our understanding of the metabolic processes involved in GBM pathogenesis. Our Mendelian Randomization analysis of 1,400 plasma metabolites has identified 69 kinds of metabolites that appear to be associated with the risk and progression of GBM, most notably TMAO levels. This metabolite’s positive association with GBM development indicates its potential role in the pathophysiological mechanisms underlying this malignancy.

TMAO is an organic compound synthesized in the liver from trimethylamine (TMA), a byproduct of gut microbiota metabolism of nutrients dense in choline and carnitine, predominantly sourced from red meat, eggs, and dairy products [26]. The compound has come into scientific focus due to its associations with a variety of diseases, most notably cardiovascular disorders [27]. Its role in oncology is gaining interest, as emerging studies suggest potential mechanisms by which TMAO may influence cancer progression [9, 28, 29]. The promotion of inflammation, a recognized hallmark of cancer, has been considered to be associated with elevated levels of TMAO [30, 31]. Chronic inflammation is known to play a role in the initiation and advancement of tumor development [32, 33]. Furthermore, TMAO is thought to modulate immune responses, potentially impacting the body’s capacity to combat tumor cells [30]. Additionally, TMAO may affect cellular signaling pathways that are crucial for cell proliferation and survival [34], as well as contribute to oxidative stress [35], which can lead to enhanced tumor growth [34]. Nevertheless, the exact mechanisms by which TMAO may influence cancer pathogenesis, including that of GBM or other solid tumors, remain inadequately defined. TMAO’s impact may be direct, through interactions with tumor cells, or indirect, by altering the tumor microenvironment or systemic metabolism.

The association between TMAO and GBM identified in this study suggests its potential utility as a non-invasive biomarker for early detection or monitoring of disease progression, which could be particularly beneficial in clinical settings where current primary diagnostic methods are invasive. Our findings suggest that dietary modifications to reduce TMAO production—a strategy potentially involving decreased consumption of red meat and other choline- or carnitine-rich foods—could be a viable approach to reducing GBM risk. This dietary advice aligns with current recommendations for the prevention of cardiovascular diseases and other conditions associated with high TMAO levels [26].

Importantly, our further research suggests a unidirectional relationship where elevated TMAO levels might contribute to the risk or progression of GBM, but the presence of GBM does not, in turn, affect TMAO levels. These findings indicate that while TMAO may play a role in the pathogenesis of GBM, the disease’s progression does not seem to exert a reciprocal effect on systemic TMAO levels. The findings reinforce the potential of plasma TMAO levels as a biomarker for GBM risk assessment. However, TMAO levels may not reflect disease progression or therapeutic response, as GBM does not appear to influence these levels. In addition, it also lacks a significant causal relationship between TMAO levels in CSF and the development of GBM.

The discrepancy between the role of TMAO levels in CSF and plasma in relation to GBM can be attributed to several factors. CSF and plasma have distinct biochemical compositions and serve different physiological functions [36]. Plasma is involved in systemic circulation and reflects metabolic processes occurring throughout the body [37]. In contrast, CSF is more localized to the brain and spinal cord, indicating that the metabolic environment in the brain might be distinct from systemic circulation [38], and thus the levels of TMAO in CSF and plasma could be influenced by different factors. Furthermore, the Blood-Brain Barrier (BBB) plays a crucial role in separating the brain’s microenvironment from the systemic circulation [39–41]. It is highly selective in what it allows to pass through [41]. This barrier could mean that while elevated TMAO levels in plasma might influence GBM risk or progression, these effects do not necessarily translate to similar alterations in CSF TMAO levels. In addition, the production and metabolism of TMAO are primarily located in the liver and influenced by gut microbiota [42–44]. Given the liver’s systemic connection and the BBB’s selective permeability, this could result in differing impacts of TMAO on GBM [45]. The findings suggest that systemic metabolic changes (reflected in plasma) might play a more significant role in GBM’s pathogenesis than localized metabolic alterations within the brain or spinal cord (reflected in CSF) [46]. In addition, the findings reinforce the potential of blood biomarkers, TMAO, in GBM risk assessment and early detection, which could be more accessible and less invasive than biomarkers in CSF.

Our study, while providing valuable insights into the association between TMAO levels and GBM, faces several limitations. The analysis is based on data from a specific population, which may not accurately represent the diverse genetic, dietary, and environmental factors influencing TMAO levels and GBM risk across different demographic groups, thus limiting the generalizability of our findings. Additionally, our study does not delve into the underlying biological mechanisms by which TMAO might influence GBM pathogenesis. Lastly, it is imperative to conduct extensive, multi-center validation studies to ascertain TMAO’s efficacy across varied demographics, thereby establishing its sensitivity, specificity, and predictive value for GBM. Developing standardized measurement protocols for TMAO, including reference ranges and assay reproducibility, is essential. Identifying the optimal clinical context for TMAO measurement—be it for screening, differential diagnosis, or monitoring—is crucial. Clinical trials will further evaluate TMAO’s integration into diagnostic and therapeutic regimes, assessing its contribution to patient outcomes. Challenges such as dietary and microbiome influences on TMAO levels necessitate comprehensive adjustments in studies, while the precision of TMAO measurement techniques must be continuously advanced and validated. Moreover, increasing clinician awareness and understanding of TMAO’s biomarker potential through education and consensus building is vital, alongside assessing the cost-effectiveness of TMAO testing for practical applicability. Transitioning TMAO from a research biomarker to a clinical tool encompasses tackling scientific, technical, and logistical hurdles, necessitating collaborative and iterative efforts to harness its full potential in enhancing GBM patient care and outcomes.

In conclusion, our extensive analysis of 1,400 plasma metabolites has successfully identified 69 metabolites that are associated with GBM. Our findings highlight the potential of plasma metabolites, particularly TMAO, as biomarkers for GBM, offering a promising avenue for early detection and risk assessment.

## Supplementary Materials

Supplementary Tables
