# Supplementary material for: Deciphering the causal relationship between plasma and cerebrospinal fluid metabolites and glioblastoma multiforme: a Mendelian Randomization study
Source: Aging (Albany NY). 2024 May 10;16(9):8306–19. doi: 10.18632/aging.205818 (PMC11131984; doi:10.18632/aging.205818)
Supplement: Supplementary Tables [file aging-16-205818-s001.pdf]

SUPPLEMENTARY TABLES

Supplementary Table 1. Two-sample MR analysis results of Trimethylamine N-oxide levels on GBM, including heterogeneity test and horizontal pleiotropy test.

Method	nsnp	b	se	*p*-val	lo_ci	up_ci	or	or_lci95	or_uci95	heterogeneity_Q_*p*-val	pleiotropy-*p*-val
MR Egger	15	1.5187	0.8595	0.1007	−0.1659	3.2033	4.5662	0.8471	24.6140	0.5742	
Weighted median	15	0.9542	0.4695	0.0421	0.0340	1.8743	2.5965	1.0346	6.5163		
Inverse variance weighted	15	1.1514	0.3367	0.0006	0.4915	1.8114	3.1627	1.6347	6.1189	0.1853	0.4306
Simple mode	15	1.0580	0.7415	0.1755	−0.3953	2.5113	2.8805	0.6735	12.3205		
Weighted mode	15	0.9992	0.6643	0.1548	−0.3028	2.3012	2.7161	0.7388	9.9859		

Supplementary Table 2. Two-sample MR analysis results of GBM on Trimethylamine N-oxide levels.

Method	nsnp	b	se	*p*-val	lo_ci	up_ci	or	or_lci95	or_uci95	heterogeneity_Q_*p*-val	pleiotropy-*p*-val
MR Egger	8	−0.0029	0.0349	0.9361	−0.0713	0.0654	0.9971	0.9312	1.0676	0.6968	
Weighted median	8	−0.0149	0.0199	0.4539	−0.0539	0.0241	0.9852	0.9475	1.0244		
Inverse variance weighted	8	−0.0217	0.0151	0.1502	−0.0514	0.0079	0.9785	0.9499	1.0079	0.7553	0.5711
Simple mode	8	−0.0181	0.0253	0.4981	−0.0676	0.0315	0.9821	0.9346	1.0320		
Weighted mode	8	−0.0161	0.0230	0.5058	−0.0611	0.0289	0.9840	0.9407	1.0293		

Supplementary Table 3. Two-sample MR analysis results of Trimethylamine N-oxide levels in CSF on GBM.

Method	nsnp	b	se	*p*-val	lo_ci	up_ci	or	or_lci95	or_uci95	heterogeneity_Q_*p*-val	pleiotropy-*p*-val
MR Egger	56	0.2287	0.4165	0.5852	−0.5876	1.0449	1.2569	0.5557	2.8431	0.0626	
Weighted median	56	0.2024	0.3155	0.5212	−0.4160	0.8207	1.2243	0.6597	2.2721		
Inverse variance weighted	56	0.1868	0.2304	0.4175	−0.2648	0.6384	1.2054	0.7674	1.8934	0.0745	0.9040
Simple mode	56	−0.8097	0.7073	0.2573	−2.1960	0.5767	0.4450	0.1112	1.7801		
Weighted mode	56	0.1891	0.5442	0.7295	−0.8774	1.2557	1.2082	0.4159	3.5103		
